# Experiences of the COVID-19 pandemic on child and adolescent psychiatric wards: multi-methods investigation

**DOI:** 10.1192/bjo.2024.783

**Published:** 2024-11-06

**Authors:** Josephine Holland, Morenike Da-Silva-Ellimah, James Roe, Richard Morriss, CAPSS Scientific Committee, Kapil Sayal

**Affiliations:** Mental Health and Clinical Neurosciences Unit, Institute of Mental Health, University of Nottingham, UK; National Institute for Health and Care Research Applied Research Collaboration (ARC) East Midlands, University of Nottingham, UK

**Keywords:** Childhood experience, carers, in-patient treatment, mental health services, patients

## Abstract

**Background:**

Child and adolescent mental health service in-patient beds are unevenly spread throughout England. Where demand outstrips bed availability, young people may be admitted at-distance or to adult psychiatric wards. The COVID-19 pandemic added pressures to already overstretched services. Understanding experiences during this period is vital to inform strategies for future emergencies.

**Aims:**

To investigate the impact of the COVID-19 pandemic on admissions to local, at-distance or adult psychiatric units, from the perspectives of young people, parents/carers and healthcare professionals.

**Method:**

Multi-methods data were collected from February 2021 to September 2022, as part of the Far Away from Home research programme. A 13-month national surveillance study collected information about admissions to general adolescent units >50 miles from home, out-of-region or to adult psychiatric units. Free-text data from respondents (*n* = 51) were analysed using content analysis. Interviews with young people (*n* = 30), parents/carers (*n* = 21) and healthcare professionals (*n* = 68) were analysed using thematic analysis.

**Results:**

Restrictions during the COVID-19 pandemic affected young people's contact with others; the requirement to self-isolate on admission and following overnight leave felt distressing, and visiting was limited. This disincentivised overnight leave, leading to some discharges being delayed and others feeling rushed and high risk. The COVID-19 pandemic also accelerated the introduction of virtual meetings, enabling community teams and families to be more involved in therapies, meetings and decision-making.

**Conclusions:**

Restrictions imposed during the COVID-19 pandemic were often negatively perceived. However, the increased use of technology was felt to be positive, widening inclusion and mitigating some negative effects of distance on admissions.

The immediate physical risks of the COVID-19 pandemic may have decreased significantly, but its effects continue to linger.^1^ Across the world, services are still working to contain the secondary crisis of psychological distress and mental health system spillover.^2^ Public health measures introduced during the COVID-19 pandemic to limit the spread of the virus, such as lockdown, quarantine and school closures and subsequent re-openings, disproportionately affected children and young people, increasing their emotional distress, mental health disorder symptoms and associated risks.^3–6^

In the early months of the pandemic, a survey of child and adolescent mental health service (CAMHS) in-patient wards found discharge delays and restrictions surrounding visits and time off the ward, but also some relaxation to rules around mobile telephones and video calling.^7^

At that time, there had been a significant drop in referrals to CAMHS in-patient services;^8^ since then, there has been an increase in the number of referrals to CAMHS, as well as a rise in the acuity and severity of symptoms.^9^ Research into the pandemic needs to not only focus on the early months, but cover the whole period, since the nature of the threat and associated restrictions will have had differential effects throughout.

## Data from the ‘Far Away from Home’ study

The ‘Far Away from Home’ study, a multi-methods programme of research investigating admissions of patients aged <18 years to CAMHS and adult in-patient wards, collected quantitative and qualitative data from February 2021 to September 2022.^10,11^ Findings from this research showed that at-distance or out-of-region admissions affect 13.7–16.9 per 100 000 young people aged 13–17 years in England, and the most common reason for these admissions was a lack of local CAMHS beds.^10^

Underpinned by an interpretivist approach, this paper presents a multi-methods study of the effects of the COVID-19 pandemic on experiences during this period. It uses two different data-sets: free-text responses to questionnaires and interviews. These data are from overlapping (some healthcare professionals (HCPs) completed the questionnaires and participated in interviews) and distinct (interviews with young people, parents/carers and HCPs who only gave responses in one part of the study) samples.

## Method

### Participants and data collection

The Far Away from Home study, through the Child and Adolescent Psychiatry Surveillance System (CAPSS), conducted a 13-month surveillance period from February 2021 to February 2022, with 6-month follow-up data collected until September 2022.^10^ During this period, consultant child and adolescent psychiatrists across England who reported seeing an eligible case were asked to complete two questionnaires for each case, at baseline (as soon after admission as possible) and at 6-month follow-up. Eligible cases were defined as adolescents (aged 13–17 years) who were admitted to a general adolescent unit (a psychiatric ward for young people aged 13–17 years) far away from home (more than 50 miles (80 km) from their home address or outside of their National Health Service (NHS) commissioning region), or to an adult psychiatric ward for psychiatric care. The questionnaires included a mixture of closed and open-ended questions. This paper focuses on free-text comments offered about the effects of COVID-19 on the admission, and the responses to other questions are presented elsewhere.^10^

The Far Away from Home qualitative interview study purposively recruited young people, parents/carers and HCPs through local participating NHS Trusts in five large regions in England. All young people and parents/carers had experience of at least one of the following: admission to a local CAMHS ward, admission to a far-away CAMHS ward or admission to an adult psychiatric ward. Across the five regions, 30 young people and 21 parents/carers were interviewed; two further young people and one parent consented to participate, but withdrew before the interview.

Alongside this, using purposive sampling through clinical networks, healthcare leaders and respondents from the surveillance study, HCPs working in CAMHS in-patient or CAMHS community settings, adult mental health services or private mental health services across England were approached to be interviewed about their experiences. In total, 68 HCPs were interviewed; three additional HCPs consented to participate, but withdrew before the interview.

The topic guides for all interviews are included in the Supplementary Material available at https://doi.org/10.1192/bjo.2024.783.

Interview schedules for each stakeholder group were developed in collaboration with the study professional, young person and parent/carer advisory groups. Interviews were conducted either virtually through Microsoft Teams for Windows or face to face. All interviews were 20–60 min long and audio recorded. Reponses to other interview questions are presented elsewhere;^11^ this paper focuses on themes found within comments about the impacts of the COVID-19 pandemic specifically.

### Positionality statement

J.H. (White British, female) and K.S. (Indian British, male) are clinical academics in child and adolescent psychiatry, M.D.-S.-E. (Black British African, female) is a clinical academic in paediatrics, R.M. (White British, male) is a clinical academic in adult psychiatry and J.R. (White British, male) is an academic researcher in mental health.

### Content analysis of questionnaire responses

The qualitative data about the impact of the COVID-19 pandemic, provided through free-text responses, were analysed using conventional content analysis as described by Hsieh and Shannon.^12^ Two researchers (J.H. and M.D.-S.-E.) completed the following steps: familiarisation with the data, initial coding, grouping the codes into categories and then combination of categories until main categories emerged. Any comments that did not include any content were excluded. Where comments contained relevant content, but did not have enough detail to interpret the meaning with certainty, two study team members reviewed these comments alongside the other questionnaire responses, to resolve ambiguity.

### Thematic analysis of interview responses

All interviews were transcribed verbatim and initially coded with NVivo 12 for Windows (Lumivero, Denver USA; https://lumivero.com/products/nvivo/) by the Far Away from Home research team. The interview data were thematically analysed using the five stages of framework analysis, as described by Ritchie and Spencer: familiarisation, identifying a thematic framework, indexing, charting, and mapping and interpretation.^13^ When coding was completed, the matrix framework was exported into Microsoft Excel, Microsoft 365 for Windows, with a row for each interviewee, column for each code and data within the cells. Concise summaries of the verbatim text were then produced in each matrix cell. The summaries were then grouped into overarching themes.

### Comparison and combination of themes/categories

The identified categories from the content analysis were treated as themes. The identified themes from both data sources, along with the initial codes and the data within them, were compared. Where the themes significantly overlapped, they were combined. Themes that were only present in one data source are also presented.

### Ethics statement

The authors assert that all procedures contributing to this work comply with the ethical standards of the relevant national and institutional committees on human experimentation and with the Helsinki Declaration of 1975, as revised in 2013. All procedures involving patients were approved by the South Birmingham Research Ethics Committee (approval numbers 20/WM/0265 and 20/WM/0314) and Section 251 Health Research Authority Confidentiality Advisory Group permission (20/CAG/0127).

### Consent statement

Within the Far Away from Home surveillance study, consultant child and adolescent psychiatrists reported information about young people. Participants did not specifically consent for their data to be shared, and so approval by the confidentiality advisory group (CAG) was applied for and granted. Medical records held by the NHS keep a record of whether a parent of a child does not want their medical notes to be used for research purposes. Parents/carers of young people can inform their respective consultant psychiatrist that they do not want their child's notes to be used in this sense. If any parent/carer had indicated such dissent, we did not use their data. The national data opt-out also enables patients to opt out of the use of their data for research purposes. Written informed consent was obtained from all interview participants.

## Results

### Questionnaire free-text responses

A total of 79 free-text comments were provided by 51 respondents to the surveillance study (child and adolescent psychiatry consultants, no further demographic information was collected). Eleven comments were excluded because they did not contain any relevant content; 68 were included in further analysis. The comments from the baseline and follow-up surveys were combined and analysed together. All extracts have been quoted verbatim.

### Interview data

All 119 interviewees, see Table 1 for demographic details, spoke about the impact of the COVID-19 pandemic on their experiences. All comments were included in the analysis.

**Table 1:** Demographic details of interview participants

	Young person (*n* = 30)	Parent (*n* = 21)
Gender	Female = 22	Female = 16
Age: mean years; range	Mean = 15.9 Range = 13–17	Mean = 49.65 Range = 37–63
Ethnicity	White: English, Welsh, Scottish, Northern Irish or British = 19	White: English, Welsh, Scottish, Northern Irish or British = 12
Admission type	At-distance = 19 Near home = 7 Adult ward = 2 Adult and Far Away = 2	At-distance = 9 Near home = 8 Adult ward = 2 Adult and Far Away = 2
	Healthcare professional = 68	
Role	Community consultant in child and adolescent psychiatry = 19 In-patient consultant in child and adolescent psychiatry = 17 In-patient consultant in adult psychiatry = 10 Mental health commissioner = 4 Nurse in adult mental health = 4 Clinical nurse lead/senior nurse/specialist nurse = 4 In-patient adult ward manager = 3 Private sector consultant in child and adolescent psychiatry = 3 In-patient child and adolescent ward manager = 2 Care coordinator = 1 Case manager = 1	

### Themes generated in both data-sets

#### Theme: how the COVID-19 pandemic affected young people's contact with others

COVID-19 affected young people's contact with others in a number of ways, including changes in access to the ward, self-isolation, visiting and leave rules (see Table 2 for quotations).

**Table 2:** Quotations to illustrate the theme: how the COVID-19 pandemic affected young people's contact with others

Quote	Subtheme	
1	Parents/carers unable to enter the ward	‘We didn't know what the set up was behind the doors, what the rooms are like or what.’ (P/C)
2	Challenges associated with periods of self-isolation	‘You mean she can't see anybody for a week or what's going on? And you were a bit nervous for her.’ (P/C)
3	Challenges associated with periods of self-isolation	‘Having to self-isolate at the beginning was so rough and it was absolutely awful.’ (YP)
4	Challenges associated with periods of self-isolation	‘isolation that was quite difficult …. because I was at the end of my corridor, so I was really far away from the nurse switchboard thing.’ (YP)
5	Challenges associated with periods of self-isolation	‘I got told I'd be isolating for a few days and ended up isolating for a week. And then they took me onto the main ward but in that week I hadn't met anyone on the main ward … then I could just hear the ward. I couldn't hear any of the good things, I could just hear incidents, I could hear the alarms, I could hear banging, screaming. And I was like I don't want to go on the ward, I don't want to go.’ (YP)
6	Challenges associated with periods of self-isolation	‘Isolation was terrible, they'd stick you down a corridor and say you have to stay in your room, you can't go anywhere. Staff would come in, in full PPE, just to say hi and leave. You didn't really get any staff interaction. So you were just kind of kept in a room like a prisoner, not able to do anything.’ (YP)
7	Challenges associated with periods of self-isolation	‘So it's not only that it's scary enough to go into a unit but in addition to that you are going to be stuck in a room on your own for the first 24 h.’ (HCP)
8	Challenges associated with periods of self-isolation	‘struggled with periods of isolation while waiting results of COVID swab’ (HCP QR)
9	Challenges associated with periods of self-isolation	‘COVID tests when you first get admitted, that delays a lot of the assessment.’ (HCP)
10	Changes to arrangements around visiting	‘friends haven't been allowed to visit her and that has been really hard on the young person.’ (P/C)
11	Changes to arrangements around visiting	‘I could only have like two people, but then my sister, my mum and my dad all wanted to come at the same time, and so they would have to like swap.’ (YP)
12	Changes to arrangements around visiting	‘We just had to stay on the premises. And it's only a small room because you know as with hospitals you know it's very difficult. So to book a room was not always that easy.’ (P/C)
13	Changes to arrangements around visiting/Changes to arrangements around leave	‘After the first couple of weeks we had to basically go outside when we went to visit him, which was good when weather was nice but when it was cold basically the only thing was to stay in the car.’ (P/C)
14	Changes to arrangements around visiting	‘The visiting was generally reduced because it was like right, this family are coming at 10 o'clock in the morning, this family are coming at 12 o'clock, so that they don't all arrive at the unit at the same time and that kind of thing. So I think it's had a huge impact.’ (HCP)
15	Changes to arrangements around leave	‘Due to a COVID outbreak, the ward was in lockdown for a period of time and their leave had to be suspended, this created significant dysregulation, risk to self and restraint episode.’ (HCP QR)
16	Changes to arrangements around leave	‘My leave got really messed up and I caught COVID when I was on the ward, and like obviously we hadn't been able to have visits as much.’ (YP)
17	Changes to arrangements around leave	‘You almost think it would have put people off leave because you think well I don't want five days of isolation in my room again, so I'll just speak to my mum on the phone.’ (HCP)
18	Changes to arrangements around leave	‘And if you was to leave and say go to home leave, to be with your parents at home, if you came back you would have to isolate for 14 days.’ (YP)
19	Changes to arrangements around leave	‘I mean we could spend eight hours in the car with her driving around [hospital town] (laughs) and that was classed as not risky yet bringing her home where actually we probably spent less time with her face-to-face in a confined space. So I think the COVID rules caused more issues than anything else.’ (P/C)

P/C, parent/carer; YP, young person; HCP, healthcare professional by interview; HCP QR, healthcare professional by questionnaire response.

##### Parents/carers unable to enter wards

Parents/carers talked about how already high anxiety levels when their child was admitted were worsened by being unable to see their young person's room (Q1).

##### Challenges associated with periods of self-isolation

Self-isolation periods were felt to be negative by all who commented (Q2–Q8). Parents’ worries about being unable to see their child's room were compounded with the knowledge the young person would be confined to it during their initial days (Q2). Young people talked of the loneliness and dark thoughts they experienced during this period (Q3–Q6). HCPs echoed concerns about young people during these periods (Q7, Q8), including the delays to assessment (Q9), and some felt that these periods of isolation were actively harmful for young people.

##### Changes to arrangements around visiting

Another way in which young people's contact with others was reduced was through changes to visiting. Parents and young people frequently discussed how visits were changed in several ways, including limits to their frequency, duration, who could visit (Q10), the number of people who could visit (Q11) and where they could occur (Q12, Q13), with families having to be coordinated to ensure visits were staggered (Q14).

##### Changes to arrangements around leave

A third way in which young people's contact with others was affected was through changes in leave. The closure of nearby businesses meant sometimes local leave periods were hard to fill during bad weather because there was little to do (Q13). When someone tested positive for COVID-19 on the ward, all visits and leave would be suspended for a period, which caused frustration to all involved (Q15, Q16). HCPs questioned why overnight leave had to be followed by a self-isolation period, and how this disincentivised it (Q17); young people (Q18) and parents also echoed this (Q19).

#### Theme: how the COVID-19 pandemic affected discharge planning

COVID-19 affected discharge planning in several ways (see Table 3 for quotations). Overall, the use of online discharge planning meetings was felt to make the process feel more collaborative (Q1). However, isolations after overnight leave meant fewer trials of overnight leave were completed before discharge, leaving some young people, parents and HCPs concerned that discharge was earlier or less tested than it should be (Q2, Q3). For others, the need for periods of isolation and less access to overnight leave meant that discharges were delayed (Q4). A third way in which discharge was affected was when young people were discharged because they tested positive for COVID-19, a situation that parents/carers found very stressful both because of concerns about the young person passing the virus to others at home and the worry about the alternative of having to isolate on the ward while unwell (Q5–Q7).

**Table 3:** Quotations to illustrate the theme: how the COVID-19 pandemic affected discharge planning

Quote	
1	‘I think that has helped support discharge planning more robustly and more quickly because more people are involved and keep involved earlier.’ (HCP)
2	‘It's certainly much more difficult to move people towards discharge if they can't have leave and visits.’ (HCP QR)
3	‘The young person's admission was short due to concerns about COVID cases rising and the distance from home.’ (HCP QR)
4	‘with lockdowns … that's definitely slowed discharges down because people just can't go in or out of the ward for ten days.’ (HCP)
5	‘If she had stayed, she would have been confined to her room with very little support because they didn't want to spread it in the unit. And that would have … she just couldn't even comprehend it, if she wouldn't have been allowed very much one-to-one support. So to be trapped in your room of an inpatient ward … ’ (P/C)
6	‘YP contracted COVID on ward, and was discharged early.’ (HCP QR)
7	‘The patient was due to have a period of leave prior to discharge but then they and their family caught COVID during this leave, so he was just discharged in absentium. This caused the patient to feel very abandoned by the discharge process.’ (HCP QR)

HCP, healthcare professional by interview; HCP QR, healthcare professional by questionnaire response; P/C, patient/carer.

### Themes generated only in questionnaire data

#### Theme: how the COVID-19 pandemic delayed admission and transfer

Within the questionnaire data but not the interviews, HCPs commented on the effect of COVID-19 on admission and transfer between wards (see Table 4 for quotations). Questionnaire respondents detailed several ways admission or transfer were delayed, including the ward being closed because of COVID-19-positive cases (Q1), delays getting negative tests before transfer, and young people testing positive for COVID-19 and therefore not being permitted to transfer (Q2).

**Table 4:** Quotations to illustrate the theme: how the COVID-19 pandemic delayed admission and transfer

Quote	
1	‘Admission was delayed by 15 days due to a COVID case on the ward.’ (HCP QR)
2	‘An adult bed was identified for the patient but then they got COVID and the bed was given to another patient.’ (HCP QR)
3	‘Our paediatric ward were not taking young people because they were full of adult COVID patients.’ (HCP QR)

HCP QR, healthcare professional by questionnaire response.

A subtheme generated within this theme was that ‘COVID-19 reduced the availability of beds’. The questionnaire respondents described how paediatric or psychiatric wards were sometimes being used for adult COVID-19 patients, and one reported the general adolescent unit was shut down (Q3).

### Themes generated only in interview data

#### Theme: the effect of the COVID-19 pandemic on working practices

See Table 5 for quotations.

**Table 5:** Quotations to illustrate the theme: the effect of the COVID-19 pandemic on working practices

Quote	Subtheme	
1	Introduction of the use of virtual meetings	‘Parents are invited to weekly ward rounds via Teams since the pandemic. This is much more convenient than before, where they had to drive to the ward for a 15 min slot.’ (HCP)
2	Introduction of the use of virtual meetings	‘I think things like COVID have been helpful in terms of you know, the drive for virtual meetings. I think that since COVID we've probably had an improvement in community teams, especially those that are a little bit further away, their involvement in the young people's care.’ (HCP)
3	Introduction of the use of virtual meetings	‘I think one of the benefits I probably came across in terms of the technology and providing video-link consultation. And some young people that I see they definitely want that to continue rather than coming to see me in clinic.’ (HCP)
4	Introduction of the use of virtual meetings	‘Obviously with remote things you can sort of attend but it's never quite the same experience.’ (HCP)
5	Introduction of the use of virtual meetings	‘You know like the digital stuff's good isn't it but that's not what this speciality is about. It's about sitting down in a room and talking to people isn't it?‘ (HCP)
6	Introduction of the use of virtual meetings	‘Meetings on the phone and online. And obviously it's difficult to kind of establish a rapport like that.’ (P/C)
7	Difficulties around staffing	‘there was a lot of bank staff members that you didn't really know and didn't really interact with you.’ (YP)
8	Difficulties around staffing	‘So staff were changing all the time, which again for somebody like X that needs consistency, was hellish because she'd wake up to different people all the time. And when she got to a point where she was having a crisis, somebody, a complete stranger coming in to try and help her was never going to get anywhere.’ (P/C)
9	Ward rule changes	‘Smoking in the gardens has been allowed since the pandemic, as previously people could go offsite and smoke, but since that wasn't possible they didn't want to restrict liberties.’ (HCP)

HCP, healthcare professional by interview; P/C, parent/carer; YP, young person.

##### Introduction of the use of virtual meetings

The effect of the COVID-19 pandemic on the introduction of virtual meetings was the most commonly coded theme within the interview data, with 46 participants referring to it. Most of those who commented were HCPs, for whom the introduction of virtual meetings meant community teams and other professionals, as well as parents, were able to be present in more meetings, increasing the collaborative feel of decision-making and reducing the impact of admissions far away from home (Q1–Q3). Some HCPs did offer some negative viewpoints, discussing how it may decrease the quality of interactions (Q4, Q5). Young people and parents echoed some of these negatives, talking about how it can be difficult to build rapport over a screen, and complex interventions such as family therapy may feel more awkward online (Q6).

##### Difficulties around staffing

A further effect on working practices that was highlighted by young people, parents and HCPs was the effect of the pandemic on ward staffing. The pressures of the pandemic affected both the availability and the resilience of staff, causing higher reliance on temporary staff, which was felt to be negative (Q7, Q8).

##### Ward rule changes

Some of the ward rules changed during the COVID-19 pandemic, young people welcomed some changes such as being able to smoke on the hospital site, but others, such as cancelled trips and group activities, were felt to have a negative impact (Q9).

#### Theme: general population effects of the COVID-19 pandemic

Another theme among the HCP interviewees (that did not emerge from the questionnaire data) focused on the effects of the COVID-19 pandemic on the wider population and those presenting to CAMHS (see Table 6 for quotations). Several interviewees commented on how the COVID-19 pandemic had an overall detrimental effect on everyone (Q1), and in some young people prompting a deterioration in some that led to the need for admission (Q2). There was a consensus that since the beginning of the pandemic there were fewer presentations with psychotic symptoms, but a higher percentage of presentations involving eating disorders and autism spectrum disorder (Q3–Q5). HCPs also discussed how referral rates during the early part of the COVID-19 pandemic went down, but then increased; there were concerns that changes in community practice with online mental health reviews had led to some presentations being more florid and severe when they were admitted (Q6, Q7).

**Table 6:** Quotations to illustrate the theme: general population effects of the COVID-19 pandemic

Quote	
1	‘It's not just X, I think it was COVID and the pandemic has affected everybody really you know, so I just hope we all can pull through to be honest.’ (P/C)
2	‘But for some children they've really deteriorated because of COVID. So we've had admissions that probably were exacerbated by COVID, by people not coping with not being able to go to school and not having a normal life.’ (HCP)
3	‘So we've continued to have a decreased level of admissions with psychosis and emotional dysregulation but we've seen a massive increase in eating disorder admissions.’ (HCP)
4	‘So we have seen more referrals for young people with autism. And young people with eating disorders. And also they have stayed longer in hospital because of COVID and because of the restrictions and the lockdown.’ (HCP)
5	‘I think we've seen an increase in referrals, particularly for eating disorders requiring nasogastric-feeding, which has led to more out of area placements because it's a specialist area.’ (HCP)
6	‘When COVID first hit there was the emptying out of the inpatient units. Suddenly inpatient units found a reason to discharge people that they had been holding on to for years. Suddenly young people didn't want to be admitted, so the admission rates dropped right down as well. So I thought that was very, very interesting and probably warrants further investigation to understand why that was able to happen.’ (HCP)
7	‘So I think COVID's impacted broadly and it's increased the number of crisis presentations and I think the ability of CAMHS teams to contain crisis has been reduced because of less face-to-face.’ (HCP)

P/C, parent/carer; HCP, healthcare professional by interview.

## Discussion

This study used two methodologies to investigate experiences of CAMHS in-patient admissions during the COVID-19 pandemic, gathering information from many participants and bringing together multiple perspectives. It is known that restrictions introduced during the COVID-19 pandemic placed a limit on the social interactions of young people within the general population. However, our findings starkly show how restrictions were amplified for young people who required psychiatric admission, with requirements for periods of total isolation in their room, limits to visits with family and loved ones, and disruption to leave. The imposition of these restrictions caused distress for young people, parents and HCPs, as well as additional complications for care transitions such as admissions, transfers and discharges. There was additional strain on units during the pandemic, with difficulties in staffing noted by all involved.

However, some positive changes resulted from the COVID-19 pandemic: the introduction of virtual meetings has increased collaboration and joint decision-making, and also mitigated some of the negative effects of admissions far away from home. These findings are in keeping with other studies published during the pandemic that found more positive than expected reports from clinicians about remote consultations.^14^ However, online reviews are not perceived as universally beneficial or practical, and there are concerns relating to access equality.^15^ Some argue for a need to co-produce guidelines for best practice in remote working, and to ensure that practitioners are adequately trained.^16^

Studies have shown a significant negative effect of isolation and loneliness on symptoms of mental disorders.^17,18^ Our findings highlight how this effect of the pandemic was particularly pronounced for young people admitted to psychiatric hospitals, since they experienced isolation from even their close family during these periods, which is particularly worrying because this population is already significantly vulnerable and unwell.^19^ A study by Hanss and colleagues^20^ suggested that content-restricted devices may be a way that technology could be safely used to combat the effects of self-isolation on patients admitted to adolescent in-patient psychiatric units. The feasibility of such devices in these settings needs further investigation.

Large surveys have shown an increase in the prevalence of probable mental disorders in young people since the pandemic,^21,22^ and our findings add to the literature, focusing on how it affected this unique group. The change in population being referred for in-patient care is also supported by wider research that has found lockdowns were associated with increased levels of depression and anxiety among adolescents and exacerbated eating disorders.^23,24^

The introduction of virtual meetings, and the positives and negatives of this,^14–16,25–27^ have been discussed. A survey by Bentham et al found that staff at a UK community CAMHS service perceived the lack of face-to-face visits during the pandemic as hindering their ability to build rapport with young people and undertake core aspects of their role, as well as creating delays.^28^ The role of virtual meetings may differ between the in-patient setting (where the young person is reviewed in-person, but some therapies and meetings may be conducted online) and an out-patient setting (where reviews may be conducted online). For the population within our study, who have complex needs and often require multi-agency working, online meetings appear to provide a helpful way to promote collaboration across networks.

### Strengths and limitations

A strength of this study is the multi-methods approach to data collection. The surveillance survey enabled participants to respond at any time that was convenient, and so allowed many responses to be collected in a relatively short period. This was combined with richer, more in-depth interview data, which allowed the interviewer to flexibly explore a participants’ responses. HCPs from every region of England were interviewed to ensure generalisability across the country. A limitation of this study is that it captured only experiences in England and did not feature those living and working in other parts of the UK. The free-text survey responses were able to capture comments from a large number of HCPs; however, this method of data collection does not allow the opportunity for the study team to respond to these comments either for clarification or to elicit more information. The large amount of data included in the analysis meant it was not possible to go into depth about individual experiences or capture the phenomenology of this topic in more specific detail. A further limitation, but also potential strength, was that responses were not segregated based on the time of capture; the questionnaire respondents were asked to comment on the effect of the COVID-19 pandemic on a particular case they were reporting, but some responded about the more general effects of COVID-19. Alongside this, the participants interviewed were asked to comment more generally on the effects of COVID-19, and therefore the data were able to capture not only specific effects on a person's admission, but also a more longitudinal view of how the COVID-19 pandemic had affected services.

### Clinical implications

The COVID-19 pandemic and associated restrictions has had an overall negative effect on the mental health of young people, which was amplified for young people admitted to CAMHS wards. However, some of the changes resulting from the pandemic, such as virtual meetings, have provided a useful tool that will continue to be used in everyday practice. For future national emergencies, the unique situation of those admitted to CAMHS in-patient units, who are young and physically healthy but mentally extremely vulnerable, should be considered when deciding how and whether public health restrictions are applied in these settings.

## Supporting information

Holland et al. supplementary material 1 Holland et al. supplementary material

Holland et al. supplementary material 2 Holland et al. supplementary material

Holland et al. supplementary material 3 Holland et al. supplementary material

Holland et al. supplementary material 4 Holland et al. supplementary material

## Data Availability

The data that support the findings of this study are available on reasonable request from the corresponding author, J.H.
