# Supplementary material for: Experiences of the COVID-19 pandemic on child and adolescent psychiatric wards: multi-methods investigation
Source: BJPsych Open. 2024 Nov 6;10(6):e197. doi: 10.1192/bjo.2024.783 (PMC11698167; doi:10.1192/bjo.2024.783)
Supplement: Holland et al. supplementary material 1 — Holland et al. supplementary material [file S205647242400783Xsup001.docx]

**Far Away from Home**

**Adult psychiatrist interview guide**

**Structure**

One 30-45 minute telephone/Zoom/MS Teams interview

**Objective**

We are interested in finding out about the experience of different kinds of inpatient admission for young people (aged 13-17) and their families as well as their benefits and disadvantages. This includes the experience of young people admitted to an adult psychiatric ward.

**Equipment**

- Participant Information Sheet
- Consent form
- Demographics questionnaire

**Pre-interview**

- Researcher has obtained informed consent from participant
- Demographic information to be obtained

**Introduction**

*Introduction:* Researcher to introduce self. Welcome and thank participant for taking part.

*Structure:* Explain set up and length of time. Participants reminded that the interview will be recorded and field notes may also be taken during the interview.

*Consent:* To researcher and study: answer questions and check understanding

Reassurance: participation is voluntary and the interview can be stopped at any time;

No pressure to answer questions: participant is in control

Interview content is confidential: will not be disclosed to young person, family or professionals, except if safeguarding issues are raised (explain clearly what this might involve, what will happen and that participant will be involved in discussion and decisions)

Ask for permission to tape: fine if not, researcher will take notes

Completion of written consent to interview. Verbal confirmation on tape recording for telephone/skype interviews.

*Study purpose:* Briefly introduce the Far Away from Home study. Purpose of today’s interview

*NB The interview guide gives an indication of the type and range of questions which will be covered in the interview. It is not a script, and the discussion will develop in response to the participant’s contribution, and will pick up and explore issues of particular relevance and salience to each case. The wording and direction of questions will be tailored to individual participant’s circumstances and phrased sensitively and appropriately according to context.*

| **Topic & Timing** | **Discussion Point** | **Prompts** |
| --- | --- | --- |
| **Case Example background and circumstance**  10 minutes | *“Can you tell me, first of all, about a recent case involving referral of a young person to an inpatient adult unit”* | Seek to establish a detailed narrative account, including  Events leading up to referral  What factors indicated that inpatient admission was necessary?  were there any other options?  Process of referral and how it was initiated  discussion with other health professionals, YP and family  How did the YP and other family members feel about being referred to unit?  Relieved, apprehensive, unnecessary, resisted etc  To what extent did you feel that the YP and family were consulted, involved in decisions about care?  Do you think YP found it helpful to be in the unit?  In what way? What did think helped them most?  Do you think adult ward staff have the skills and experience necessary to care for YP under the age of 18?  Were there any negative aspects or consequences of the admission?  e.g. difficulties of YP’s integration with adult patients and ward activities, safeguarding issues, impact on other patients, consequences for staff time and work load  How did you find communication and contact with other health professionals, including CAMHS, involved in YP’s care?  How would you assess the outcome of the YP’s admission?  Do you think (the ward) was an appropriate place for someone of YP’s age?  If not, what would have been the best place? |
| **Impact of admission**  5 minutes | *“What are the consequences for YP of being admitted to an adult ward: negative and positive?”* | e.g. re experience of being an inpatient, contact with family, contact with familiar services and health professionals, ease of reintegration into family, school, community, etc  Do inpatient admissions pose any issues regarding continuity of care and the YP’s engagement with CAMHS following discharge home?  Do you think that some YP might prefer admission to an adult, rather than a children’s unit, or is this always inappropriate or less than ideal? (Explore) |
| **Experience and Perceptions**  10 minutes | YP was admitted to an adult ward. How often does this happen? | Under what circumstances (choice, necessity etc)  What is your view about young people being admitted to adult wards?  How regularly does this happen?  Who refers these patients?  Do you receive appropriate information in the documentation?  Do you have a protocol for managing young people on adult wards? If yes, useful or not? If no, how do you plan?  What involvement do you have in planning and managing the process of these admissions?  To what extent do you feel that young people (aged 13-17 years) can be properly cared for on adult wards?  How confident do you feel in engaging with these young people?  What specific of issues do you experience when a young person is admitted to your adult ward?  How does admitting a young person to an adult ward impact on the treatment that can be offered?  How do you feel admission to an adult ward affects the patient and family experience?  How are CAMHS consultants / GPs involved in joint working in such cases? Barriers? How could this be improved?  What involvement do you have in planning continuity of care / post discharge care? Who else is involved in this? How easily is this achieved? How could it be improved?  Where referrals are not accepted, what are the reasons? Is an alternative offered?  What do you think needs to happen to reduce admissions of young people to distant adolescent units / adult psychiatric wards (as appropriate)?  What is the frequency of having a young person under 18 admitted to your ward?  What are the most difficult aspects of having a young person under 18 on the ward?  Was there anything positive in the experience of having young person under 18 on the ward?  Did you find your Child and Adolescent Psychiatry colleagues helpful or unhelpful? In what ways were they helpful or unhelpful?  What are your thoughts about the effect of the admission to an adult ward on the young person and their family? |
| **Comparisons with other admissions**  5 minutes | *“How does your experience of cases such as these differ during the COVID-19 pandemic* |  |
| **Reflection**  5 minutes | *“Do you think it should be possible for people in YP’s situation to avoid inpatient admission, and be treated in the community?”* | what would be required to enable this to happen? |
| **Policy/Guidelines**  10 minutes | *“How do current practice guidelines and policy drivers inform your care of young people like XX(YP)?”* | Are there any changes in current policy/clinical guidelines that you feel would improve current care of YP in psychiatric services?  Are there any changes you would like to see in the organisation and resources available to you in managing the care of YP with severe mental health problems? |
| **Ending & Sum Up**  5 minutes | *“Is there anything else you have to add to what we have been talking about? Anything that is important that we haven’t covered already?”* |  |

Thank you very much for your help with our research and for taking part in this interview.

*Explain timeline and output of the study and how access to results will be provided.*
