# Supplementary material for: Experiences of the COVID-19 pandemic on child and adolescent psychiatric wards: multi-methods investigation
Source: BJPsych Open. 2024 Nov 6;10(6):e197. doi: 10.1192/bjo.2024.783 (PMC11698167; doi:10.1192/bjo.2024.783)
Supplement: Holland et al. supplementary material 2 — Holland et al. supplementary material [file S205647242400783Xsup002.docx]

**Far Away from Home**

**Child and adolescent psychiatrist interview guide**

**Structure**

One 30-45 minute telephone/Zoom/MS Teams interview

**Objective**

We are interested in finding out about the experience of different kinds of inpatient admission for young people (aged 13-17) and their families as well as their benefits and disadvantages.

**Equipment**

- Participant Information Sheet
- Consent form
- Demographics questionnaire

**Pre-interview**

- Researcher has obtained informed consent from participant
- Demographic information to be obtained

**Introduction**

*Introduction:* Researcher to introduce self. Welcome and thank participant for taking part.

*Structure:* Explain set up and length of time. Participants reminded that the interview will be recorded and field notes may also be taken during the interview.

*Consent:* To researcher and study: answer questions and check understanding

Reassurance: participation is voluntary and the interview can be stopped at any time;

No pressure to answer questions: participant is in control

Interview content is confidential: will not be disclosed to young person, family or professionals, except if safeguarding issues are raised (explain clearly what this might involve, what will happen and that participant will be involved in discussion and decisions)

Ask for permission to tape: fine if not, researcher will take notes

Completion of written consent to interview. Verbal confirmation on tape recording for telephone/skype interviews.

*Study purpose:* Briefly introduce the Far Away from Home study. Purpose of today’s interview

*NB The interview guide gives an indication of the type and range of questions which will be covered in the interview. It is not a script, and the discussion will develop in response to the participant’s contribution, and will pick up and explore issues of particular relevance and salience to each case. The wording and direction of questions will be tailored to individual participant’s circumstances and phrased sensitively and appropriately according to context.*

| **Topic & Timing** | **Discussion Point** | **Prompts** |
| --- | --- | --- |
| **Case Example background and circumstance**  10 minutes | *“Can you tell me, first of all, about a recent case involving referral of a young person to an inpatient unit: this could be locally, at some distance from home, or to an adult ward”* | Seek to establish a detailed narrative account, including  Events leading up to referral  What factors indicated that inpatient admission was necessary?  were there any other options?  Process of referral and how it was initiated  was there more than one option of inpatient unit?  was the YP admitted to R’s preferred unit?  if not, why not?  How did YP and R/other family members feel about being referred to unit?  Relieved, apprehensive, unnecessary, resisted etc  To what extent did you feel that the YP and family were consulted, involved in decisions about care?  What do you perceive to be the actual, appropriate and desired involvement of family in discussion and decisions about care?  What kind of contact, if any, did you maintain with 1. the YP and 2. the family during the period of admission?  what were the consequences of being able/  not able to maintain such contact?  Do you think YP found find it helpful to be in the unit?  In what way? What did think helped them most?  Were there any negative aspects or consequences of the admission?  How would you assess the outcome of the YP’s admission?  How would you manage a similar episode affecting this YP in future?  Do you think (the unit) was an appropriate place for someone of YP’s age?  If not, what would have been the best place? |
| **Impact of admission**  5 minutes | *“Are the impacts / consequences for young people similar for each type of admission*  *Then if not how do they vary (adult, distant, local): negative and positive?”* | e.g. re experience of being an inpatient, contact with family, contact with familiar services and health professionals, ease of reintegration into family, school, community, etc  Do inpatient admissions pose any issues regarding continuity of care and the YP’s engagement with CAMHS following discharge home?  Do you think it can ever be helpful for some YP to be admitted to a unit at some distance from home? (If so, in what ways)  Do you think that some YP might prefer admission to an adult, rather than a children’s unit, or is this always inappropriate? (Explore) |
| **Experiences and Perceptions**  10 minutes | “YP was admitted to a local/distant YP unit/adult ward. How often does this happen?” | Under what circumstances (choice, necessity etc)  What about (the other two options): do you ever refer YP to either of these?   - under what circumstances?   How do you feel that admission to a local adolescent unit differs from a distance adolescent unit (as appropriate)? Pros / cons?  What is your view about young people being admitted to distance adolescent units?  How regularly do you experience this?  Who refers these patients?  Do you receive appropriate information in the documentation?  Do you have a protocol for managing young people at distance from home? If yes, useful or not?  What involvement do you have in planning and managing the process of these admissions? Who else is involved?  Are there any particular issues that you have faced in these circumstances?  How do you feel admission to a ward at distance from home affects the patient and family experience?  To what extent is there joint working with CAMHS consultants / GPs in the young people’s local areas? How is this achieved / barriers to this? How could it be improved?  To what extent (if at all) does admission to a distance adolescent unit influence the success of treatment / the experience of patients and family?  How do you plan the continuity of care / post discharge care? Who else is involved in this? How easily is this achieved? How could it be improved?  Where referrals are not accepted, what are the reasons? Is an alternative offered?  What do you think needs to happen to reduce admissions of young people to distant adolescent units / adult psychiatric wards (as appropriate)? |
| **Comparisons with other admissions**  5 minutes | *“How has your experience of cases such as these differed during the COVID-19 pandemic”* |  |
| **Reflection**  5 minutes | *“Do you think it should be possible for people in YP’s situation to avoid inpatient admission, and be treated in the community?”* | If far away / adult or in general?  what would be required to enable this to happen? |
| **Policy/Guidelines**  5 minutes | *“How do current practice guidelines and policy drivers inform your care of young people like XX(YP)?”* | Do you think it should be possible for people in YP’s situation to avoid inpatient admission, and be treated in the community?   - - what would be required to enable this to happen?   Are there any changes in current policy/clinical guidelines that you feel would improve current care of YP in psychiatric services?  Are there any changes you would like to see in the organisation and resources available to you in managing the care of YP with severe mental health problems? |
| **Ending & Sum Up**  5 minutes | *“Is there anything else you have to add to what we have been talking about? Anything that is important that we haven’t covered already?”* |  |

*If not already clear/as appropriate, and using the described case as a point of reference and comparison establish:*

Thank you very much for your help with our research and for taking part in this interview.

*Explain timeline and output of the study and how access to results will be provided.*
