# Supplementary material for: Experiences of the COVID-19 pandemic on child and adolescent psychiatric wards: multi-methods investigation
Source: BJPsych Open. 2024 Nov 6;10(6):e197. doi: 10.1192/bjo.2024.783 (PMC11698167; doi:10.1192/bjo.2024.783)
Supplement: Holland et al. supplementary material 3 — Holland et al. supplementary material [file S205647242400783Xsup003.docx]

**Far Away from Home**

**Parent interview guide**

**Structure**

One 30-45 minute telephone/Zoom/MS Teams interview

**Objective**

We are interested in finding out about the experience of different kinds of inpatient admission for young people and their families.

**Equipment**

- Participant Information Sheet
- Consent form
- Demographics questionnaire

**Pre-interview**

- Researcher has obtained informed consent from participant
- Demographic information to be obtained

**Introduction**

*Introduction:* Researcher to introduce self. Welcome and thank participant for taking part.

*Structure:* Explain set up and length of time. Participants reminded that the interview will be recorded and field notes may also be taken during the interview.

*Consent:* To researcher and study: answer questions and check understanding

Reassurance: participation is voluntary and the interview can be stopped at any time;

No pressure to answer questions: participant is in control

Interview content is confidential: will not be disclosed to family or professionals, except if safeguarding issues are raised (explain clearly what this might involve, what will happen and that participant will be involved in discussion and decisions)

Ask for permission to tape: fine if not, researcher will take notes

Completion of written consent to interview

(If appropriate) nomination of family carer for study (may be discussed at end of interview)

*Study purpose:* Briefly introduce the Far Away from Home study. Purpose of today’s interview

*NB The interview guide gives an indication of the type and range of questions which will be covered in the interview. It is not a script, and the discussion will develop in response to the participant’s contribution, and will pick up and explore issues of particular relevance and salience to each case. The wording and direction of questions will be tailored to individual participant’s circumstances and phrased sensitively and appropriately according to context.*

| **Topic & Timing** | **Discussion Point** | **Prompts** |
| --- | --- | --- |
| **Background**  5 minutes | Can you tell me, first of all, about your recent experience of [name of YP] admission to [name of hospital/unit]? | Seek to establish a detailed narrative account, including   - Events leading up to admission - Process of referral and how it was initiated - YP and family members feelings about referral to unit e.g. relieved, apprehensive, unnecessary, resisted etc - Feelings around control, speed of process - Impact on work, what did you have to do immediately? - Extent to which respondent (R) felt they were consulted, involved in decisions about care - Expectations of the unit, before YP was admitted Appropriate and desired involvement of family in discussion and decisions about care - whether there was a choice of options for YP and whether this was important |
| **Experience**  10 minutes | Can you tell me what happened, once [YP] was admitted? What was it like for them being a patient at [name of hospital/unit]? | Experience of environment, other patients, staff, information, communication, support, treatment, involvement in decisions, length of stay, visits/support from family and friends  Experience of keeping in touch with YP and impacts on providing support e.g. privacy/environment, difficulties visiting, time, distance, child care, additional costs  Costs, travel and impacts of this not just at the time but consequently  Things the YP found helpful/unhelpful in the unit  e.g. appropriateness of unit for their age  R’s their involvement in YP’s care  Information about what was happening and treatment plan, access and communication with staff  Discussion and involvement in decision making |
| **Other experiences**  5 minutes | Was this the first time YP had been admitted as an inpatient? | If no – explore previous admissions, as above.  Difference to other admissions  Cross over from child to adult services |
| **Post admission**  10 minutes | Since YP came home after being in [name of hospital/unit], how have things been? Can you tell me how things are just now? | - Health (any treatment) - Length of treatment - Home circumstances - School - Relationships - YP’s plans and aspirations for the future and any concerns about this |
| **COVID-19**  5 minutes | *If during current admission*  How was your experience during the COVID-19 pandemic? | Difference with other admissions if relevant  Visiting experience  Impact on access |
| **Reflection**  5 minutes | Looking back, what do you think about the time YP spent in [name of hospital/unit] | Helpful/unhelpful  positive or negative impact  e.g. Friends, family, support groups, activities etc: relative importance, compared to admission and professional help  Ways that could have improved the experience  advice for others in same situation |
| **Ending & Sum Up**  5 minutes | Is there anything else you have to add to what we have been talking about? Anything that is important that we haven’t covered already? |  |

Thank you very much for your help with our research and for taking part in this interview.

Is there anything else you have to add to what we have been talking about? Anything that is important that we haven’t covered already?

*Explain timeline and output of the study and how access to results will be provided.*

**Additional Questions/Prompts**

How would you describe your own well-being and that of your other family members during the time your child was in hospital?

What contribution did you have in the decision to admit your child to a local adolescent unit / distant adolescent unit / adult psychiatric ward?

What contribution did you make to the decision-making about the treatment your child received?

How satisfied were you with the treatment your child received in hospital?

How well were you kept informed of your child’s progress when he/she was in hospital?

How satisfied were you with the decisions about your child’s ongoing care? To what extent were you involved in deciding this?

What do you think needs to happen to reduce admissions of young people to distant adolescent units / adult psychiatric wards (as appropriate)?

Did the fact that the admission was going to be out-of-area/in an adult ward influence your willingness to agree to it?

Was there any positive aspect to your son/daughter being in an out-of-area admission /on an adult ward?

What were the financial implications of having your son/daughter in an out-of-area admission?

Were you able to keep in contact with the inpatient team to keep updated about your son/daughter’s progress? What difficulties did you have to face?
