# Supplementary material for: Experiences of the COVID-19 pandemic on child and adolescent psychiatric wards: multi-methods investigation
Source: BJPsych Open. 2024 Nov 6;10(6):e197. doi: 10.1192/bjo.2024.783 (PMC11698167; doi:10.1192/bjo.2024.783)
Supplement: Holland et al. supplementary material 4 — Holland et al. supplementary material [file S205647242400783Xsup004.docx]

**Far Away from Home**

**Young person’s interview guide**

**Structure**

One 30-45 minute telephone/Zoom/MS Teams interview

**Objective**

We are interested in finding out about young people’s experience of different kinds of inpatient admission.

**Equipment**

- Participant Information Sheet
- Consent form
- Demographics questionnaire

**Pre-interview**

- Researcher has obtained informed consent from participant
- Demographic information to be obtained

**Notes**

- Feedback from YP’s PPI: booking interviews - allow people time to adjust and transition from hospital to home. Be mindful that some participants may be anxious about video calls.
- Suggested questions from PPI below
- Feedback from parent PPI: YP may be reluctant to talk about experiences due to trauma.

**Introduction**

*Introduction:* Researcher to introduce self. Welcome and thank participant for taking part.

*Structure:* Explain set up and length of time. Participants reminded that the interview will be recorded and field notes may also be taken during the interview.

*Consent:* To researcher and study: answer questions and check understanding

Reassurance: participation is voluntary and the interview can be stopped at any time;

No pressure to answer questions: participant is in control

Interview content is confidential: will not be disclosed to family or professionals, except if safeguarding issues are raised (explain clearly what this might involve, what will happen and that participant will be involved in discussion and decisions)

Ask for permission to tape: fine if not, researcher will take notes

Completion of written consent to interview

(If appropriate) nomination of family carer for study (may be discussed at end of interview)

*Study purpose:* Briefly introduce the Far Away from Home study. Explain the purpose of today’s interview and that we will be asking them about their recent experience of being a patient at [name of hospital/ward].

*NB The interview guide gives an indication of the type and range of questions which will be covered in the interview. It is not a script, and the discussion will develop in response to the participant’s contribution, and will pick up and explore issues of particular relevance and salience to each case. The wording and direction of questions will be tailored to individual participant’s circumstances and phrased sensitively and appropriately according to context.*

| **Topic & Timing** | **Discussion Point** | **Prompts** |
| --- | --- | --- |
| **Contextual background**  (5 mins) |  | - Contextual information about participant e.g. family, friends, work/study to get participant talking and to feel comfortable |
| **Circumstances leading to admission**  10 minutes | “Can you tell me, first of all, about your recent experience of being a patient at [insert name of hospital/ward]?”  What was it like at the beginning of admission?  How did it change? | - Events leading up to admission - Process of referral and how it was initiated - How did YP feel about being referred to unit   - Relieved, apprehensive, unnecessary - To what extent did she/he feel consulted, involved in decisions about care   - Did YP feel she/he had a choice? Was this important? - What were her expectations of the unit, before she/he was admitted? |
| **Experiences during admission**  10 minutes | “Can you tell me what happened, once you were admitted? What was it like being a patient at [insert name of hospital/ward]?”  “How was it being away from home?” | - Environment   - Other patients   - Staff   - Information, communication, support, trust   Treatment   - Type, effect, acceptability, Involvement in decisions   Length of stay   - Visits/support from family and friends - any issues, difficulties experienced in visiting?   Did you find it helpful to be in the unit?   - In what way? What did you like most? - Were there any things you did not like?   Do you think that being admitted to [insert name of hospital/ward] helped you get better? |
| **Type of unit specific** | “How did you feel about being admitted to a / distant adolescent unit / adult psychiatric ward?” | - Positive / negative experiences (e.g. homesick, stigma) - How much input did you have in the decision to admit you to this unit? - Was [insert name of hospital/ward] an appropriate place for someone of your age? - Do you think [insert name of hospital/ward] was the best place for you at the time? - Was there a better alternative? - Did the fact that the admission was going to be out-of-area/in an adult ward influence your willingness to agree to it? - What were the most difficult aspects of being in an out-of-area admission/on an adult ward? - Was there any positive aspect to being in an out-of-area admission /on an adult ward? - If your treating team recommended another admission in the future and you knew that it is going to be out-of-area/on an adult ward, would you agree to it? |
| **Comparisons with other admissions**  5 minutes | “Was this the first time you had been admitted as an inpatient?” | - If no – explore previous admissions, as above. - Covid-19 experience |
| **Circumstances post admission**  5 minutes | “Since you came home after being in [insert name of hospital/ward], how have things been? Can you tell me how things are just now?” | - Health (any treatment; what does YP think of this; could it be improved/made more acceptable?) - Home circumstances - School - Relationships - Do you have any idea about what you want to do in future? - Aspirations re health, job etc |
| **Reflection**  10 minutes | “Looking back, what do you think about the time you spent in [insert name of hospital/ward]” | - Helpful/unhelpful - good/positive or bad or negative effects? - anything else that helped you get better   Friends, family, support groups, activities etc : relative importance, compared to admission and professional help   - What do you think needs to happen to reduce admissions of young people to distant adolescent units / adult psychiatric wards (as appropriate)? - Can you think of any ways that the experience of young people in your situation when you were admitted to [insert name of hospital/ward] could be improved: - Finally, what advice would you give someone who was in your situation, just before you went to [insert name of hospital/ward]? |
| **Ending & Sum Up**  5 minutes | “Is there anything else you have to add to what we have been talking about? Anything that is important that we haven’t covered already?” | - Thank them for their participation - Explain timeline and output of the study and how access to results will be provided. |
